# Grf10 regulates the response to copper, iron, and phosphate in *Candida albicans*

**DOI:** 10.1093/g3journal/jkad070

**Published:** 2023-03-26

**Authors:** Tanaporn Wangsanut, Sylvia J Y Arnold, Safia Z Jilani, Sarah Marzec, Robert C Monsour, Ronda J Rolfes

**Affiliations:** Department of Biology, Georgetown University, Washington, DC 20057, USA; Department of Microbiology, Faculty of Medicine, Chiang Mai University, Chiang Mai 50200, Thailand; Department of Biology, Georgetown University, Washington, DC 20057, USA; Department of Chemistry, Georgetown University, Washington, DC 20057, USA; Center for Sustainable Nanotechnology, University of Minnesota, Minneapolis, MN 55455, USA; Department of Biology, Georgetown University, Washington, DC 20057, USA; Department of Biology, Georgetown University, Washington, DC 20057, USA; Morsani College of Medicine, University of South Florida, Tampa, FL 33602, USA; Department of Biology, Georgetown University, Washington, DC 20057, USA

**Keywords:** *GRF10*, transcriptional regulation, phosphate, copper, *Candida albicans*

## Abstract

The pathogenic yeast, *Candida albicans*, and other microbes must be able to handle drastic changes in nutrient availability within the human host. Copper, iron, and phosphate are essential micronutrients for microbes that are sequestered by the human host as nutritional immunity; yet high copper levels are employed by macrophages to induce toxic oxidative stress. Grf10 is a transcription factor important for regulating genes involved in morphogenesis (filamentation, chlamydospore formation) and metabolism (adenylate biosynthesis, 1-carbon metabolism). The *grf10*Δ mutant exhibited resistance to excess copper in a gene dosage-dependent manner but grew the same as the wild type in response to other metals (calcium, cobalt, iron, manganese, and zinc). Point mutations in the conserved residues D302 and E305, within a protein interaction region, conferred resistance to high copper and induced hyphal formation similar to strains with the null allele. The *grf10*Δ mutant misregulated genes involved with copper, iron, and phosphate uptake in YPD medium and mounted a normal transcriptional response to high copper. The mutant accumulated lower levels of magnesium and phosphorus, suggesting that copper resistance is linked to phosphate metabolism. Our results highlight new roles for Grf10 in copper and phosphate homeostasis in *C. albicans* and underscore the fundamental role of Grf10 in connecting these with cell survival.

## Introduction

*Candida albicans* is a ubiquitous commensal of human microbiome and a major human fungal pathogen. In health and disease, *C. albicans* resides in and infects multiple niches, including the gastrointestinal tract, skin, mouth, and the female reproductive tract (Odds 1987). Morphological plasticity allows *C. albicans* to adapt to each niche; thus, the ability to change morphologies and physiology increases its survival and virulence (Noble *et al.* 2017). In addition to morphological plasticity, metabolic flexibility is essential for *C. albicans* during commensalism, infection, and disease progression. Versatile metabolic adaption of *C. albicans* allows the fungus to assimilate the available micronutrients that vary depending upon specific host niches and host immunological defense (Miramón and Lorenz 2017).

Complex transcriptional regulatory networks control morphological switching and metabolic adaptation in *C. albicans* (reviewed in (Brown *et al.* 2014; Noble *et al.* 2017). We previously characterized the transcription factor Grf10 as an important regulator of both morphogenesis and metabolism. Grf10 is necessary for filamentation and filamentation-related processes such as chlamydospore and biofilm formation and for virulence (Chauvel *et al.* 2012; Nobile *et al.* 2012; Romanowski *et al.* 2012; Ghosh *et al.* 2015; Rodriguez *et al.* 2020). Grf10 and Bas1 regulate expression of the *ADE* regulon, which includes purine nucleotide biosynthetic genes (*ADE* genes), a nucleoside permease-encoding gene (*NUP*), and 1-carbon metabolic genes which are important for yeast growth when limited for adenine (Homann *et al.* 2009; Wangsanut *et al.* 2017). Functional characterization demonstrated that Grf10 separately controls morphogenesis and adenine-regulated transactivation (Wangsanut *et al.* 2018). However, other genes regulated by Grf10 have not yet been identified.

Grf10 is linked to copper metabolism because the *grf10*Δ mutant is able to grow on high levels of copper (Homann *et al.* 2009), suggesting a role in copper uptake or storage. Transition metals such as copper and iron are nonorganic micronutrients that are essential to cell survival and infection processes (Gerwien *et al.* 2018). Through nutritional immunity, the host sequesters trace elements away from the microbe to reduce pathogenicity during infection, or alternatively the host overwhelms the fungal cells’ ability to handle toxic levels of copper (reviewed in Hodgkinson and Petris 2012; Hood and Skaar 2012; Ding *et al.* 2014; Garcia-Santamarina and Thiele 2015; Besold *et al.* 2016; Mackie *et al.* 2016). Therefore, understanding the role of Grf10 in copper homeostasis can shed light on the host–pathogen interaction during the infection period and potentially lead to clinical significance.

Here, we focused on this copper resistance phenotype and found that growth rates were affected by gene dosage and that resistance did not extend to other metals. Prolonged incubation of the *grf10*Δ mutant under high copper levels triggered filamentous growth. We also showed that the conserved residues D302 and E305, previously shown to be necessary for activity by Grf10 during adenine limitation and filamentation (Wangsanut *et al.* 2018), are also critical for Grf10 to regulate copper toxicity. Examination of gene expression differences by RNA sequencing (RNA-seq) led to the identification of genes involved in copper, iron, and phosphate uptake and sequestration/storage. Using inductively coupled plasma mass spectrometry (ICP-MS), we found that the accumulation of copper and other metals in the *grf10*Δ mutant was no different from the wild type (WT) in low or high copper. However, we found lower accumulation of phosphorus by ICP-MS, in agreement with decreased expression of genes involved in phosphate uptake and polyphosphate synthesis. This study expands the repertoire of Grf10 target genes to include genes involved in copper, iron, and phosphate metabolism and further emphasizes that Grf10 controls multiple pathways in *C. albicans*.

## Materials and methods

### *C. albicans* strains and culturing conditions

The strains of *C. albicans* used in this study are listed in Table 1. Strains DAY185 and DAY286 were obtained from A. Mitchell (Davis *et al.* 2000, 2002), strain JC1928 was obtained from J. Quinn (Ikeh *et al.* 2016), and strains OHWT and TF021 were obtained from the Fungal Genetics Stock Center (Homann *et al.* 2009). RAC strain series were previously described (Ghosh *et al.* 2015; Wangsanut *et al.* 2017, 2018). Evolved (EV) strains were derived from WT (DAY286) by selection on 10 mM CuSO_4_ as papillae growth; 3 colonies from separate papillae were cultured in YPD and stored at −80°C.

**Table 1.** *C. albicans* strains.

Name	Genotype	Reference
DAY185	*ura3*Δ::λ*imm434/ura3*Δ::λ*imm434 his1::hisG::pHIS1/his1::hisG ARG4::URA3::arg4::hisG/arg4::hisG*	(Davis *et al.* 2000)
DAY286	*ura3*Δ::λ*imm434/ura3*Δ::λ*imm434 his1::hisG/his1::hisG ARG4::URA3::arg4::hisG/arg4::hisG*	(Davis *et al.* 2002)
RAC117	*ura3*Δ::λ*imm434/ura3*Δ::λ*imm434 arg4*::*hisG/arg4*::*hisG his1*::*hisG/his1*::*hisG grf10*Δ::*ARG4/grf10*Δ::*URA3*	(Ghosh *et al.* 2015)
RAC120	*ura3*Δ::λ*imm434/ura3*Δ::λ*imm434 arg4*::*hisG/arg4*::*hisG his1*::*hisG/his1*::*hisG grf10*Δ::*ARG4/grf10*Δ::*URA3::<GRF10, HIS1>*	(Ghosh *et al.* 2015)
OHWT	*arg4*Δ/*arg4*Δ *leu2*Δ/*leu2*Δ*::CmLEU2 his1*Δ*/his1*Δ*::CdHIS1 URA3/ura3*Δ *IRO1/iro1*Δ	(Homann *et al.* 2009)
TF021	*arg4*Δ/*arg4*Δ *leu2*Δ/*leu2*Δ *his1*Δ/*his1*Δ *URA3*/*ura3*Δ *IRO1*/*iro1*Δ *grf10*Δ::*CdHIS1*/*grf10*Δ::*CmLEU2*	(Homann *et al.* 2009)
JC1928	*arg4*Δ/*arg4*Δ *leu2*Δ/*leu2*Δ *his1*Δ/*his1*Δ *URA3*/*ura3*Δ *IRO1*/*iro1*Δ *pho4*Δ::*CdHIS1*/*grf10*Δ::*CmLEU2* CIp10	(Ikeh *et al.* 2016)
RAC256	*arg4*Δ/*arg4*Δ *leu2*Δ/*leu2*Δ *his1*Δ/*his1*Δ *URA3*/*ura3*Δ *IRO1*/*iro1*Δ *grf10*Δ::*CdHIS1*/*grf10*Δ::*CmLEU2::<GRF10, SAT1 flipper>*	(Wangsanut *et al.* 2017)
RAC259	*ura3*Δ::λ*imm434/ura3*Δ::λ*imm434 arg4*::*hisG/arg4*::*hisG his1*::*hisG/his1*::*hisG grf10*Δ::*ARG4/grf10*Δ::*URA3::<GRF10*^D302A^*, HIS1>*	(Wangsanut *et al.* 2018)
RAC260	*ura3*Δ::λ*imm434/ura3*Δ::λ*imm434 arg4*::*hisG/arg4*::*hisG his1*::*hisG/his1*::*hisG grf10*Δ::*ARG4/grf10*Δ::*URA3::<GRF10*^E305A^, *HIS1>*	(Wangsanut *et al.* 2018)
EV1	derived from DAY286 with aneuploidy	(this study)
EV2	derived from DAY286 with aneuploidy	(this study)

All strains were started from frozen glycerol stocks on solid YPD medium (Sherman 1991), grown at 30°C for 2 days, and maintained at room temperature for 10 days. YPD was supplemented with 10–13 mM CuSO_4_ as described in the text, selecting the optimum concentration under the different growth conditions to see the phenotype. Solid and liquid YPD were also supplemented with 200–1000 mM CaCl_2_, 1.0–2.5 mM CoCl_2_, 0.3–5 mM FeCl_3_ at pH 4.0, 1.0–15 mM MnCl_2_, 0.5–5 mM ZnSO_4_, or 0.32 *µ*g/ml spermidine, as indicated in the text (Ikeh *et al.* 2016). Synthetic dextrose (SD) medium with 10 mM potassium phosphate or lacking phosphate (Sherman 1991) was supplemented with 0.8 mg/ml arginine.

### Spot assays

*C. albicans* strains were grown overnight in 5-ml YPD broth at 30°C and diluted to an OD_600_ of 0.2 using sterile deionized water or were inoculated to 0.2 OD_600_ and allowed to grow to log phase and then diluted to OD_600_ of 0.2 as indicated in the figure legends. Samples were serially diluted 1:10 in sterile water, and 5 *µ*l of each dilution was spotted onto solid medium, as indicated in each figure. Samples were incubated at 30°C and photographed at 16–18 h or daily for 7 days as indicated, using an ImageQuant Imager. At least 3 biological replicates were performed for each spot assay.

### Growth rate and doubling time determination

*C. albicans* strains were grown overnight at 30°C in 5-ml YPD broth and inoculated into 96-well plate containing 200 *µ*l of YPD or YPD supplemented with the specified concentration of metal/cation at an OD_600_ of 0.01 (Schwartz *et al.* 2013). The cultures were grown at 30°C with orbital shaking in a thermo-controlled GloMax plate reader. The OD_600_ of each well was measured every 30 min for 24 or 48 h, and background OD_600_ absorption from wells containing sterile supplemented media was subtracted from each sample. Doubling times for each strain at each growth condition were calculated from the growth curve, and standard deviation was calculated in Excel. Three biological replicates with 3 technical replicates were measured and averaged for each *C. albicans* strain and growth medium.

### RNA-seq and data analysis

Two separate RNA-seq experiments were performed, both using 3 cultures of each *C. albicans* strains WT (OHWT) and *grf10*Δ (TF021) grown in 30°C. The first investigated the differences in gene expression between 1 and 4 h (#1) and the second between normal and excess copper conditions (#2).

#### Growth conditions

(#1) Strains were grown in YPD broth at 30°C for 1 and 4 h and harvested as described (Kadosh and Johnson 2005; Bruno *et al.* 2010). Briefly, a saturated overnight culture (OD_600_ of ∼10) in YPD medium was used to inoculate a 1:10 dilution for 1-h cultures or a 1:50 dilution for 4-h cultures, into 5 ml of fresh YPD medium, and cultures were grown in a tube roller at 30°C in YPD medium. Cultures were quickly chilled in an ice water bath, cells were pelleted by centrifugation, and pellets were immediately stored at −80°C. (#2) For the high copper experiment, we followed the experimental design as described (Schwartz *et al.* 2013). Strains were grown overnight in 5-ml YPD at 30°C, inoculated into 100 ml of YDP medium at an OD_600_ of 0.2, and grown at 30°C with orbital shaking until an OD_600_ of ∼1 (∼4 h). The 100-ml cultures were split into 2 50-ml portions, 12 mM CuSO_4_ was added to one of the cultures, and both were grown at 30°C for 30 min; the cells were harvested by pelleting for 10 min, and stored at −80°C until RNA extraction.

#### RNA extraction, library preparation, and RNA-sequencing

The RNA-seq data set has been deposited at Gene Expression Omnibus (Edgar *et al.* 2002; Barrett *et al.* 2013) with the SuperSeries record number GSE223218 (Wangsanut et al. 2023).

(#1) RNA was extracted from frozen pellets using the RiboPure Yeast Kit (Ambion). Copy DNA (cDNA) libraries were prepared from 1 *µ*g of DNase I-treated RNA sample using TruSeq Stranded mRNA Sample Prep Kit, following the manufacturer's directions (Illumina, RS-122-2101 Set A). RNA samples were checked for quality using an Agilent Bioanalyzer, and DNA concentration of the cDNA libraries was determined using Qubit. The cDNA libraries (10 nM) were prepared for sequencing using the MiSeq Reagent Kit, following the manufacturer's instructions (Illumina, MS-102-3001), and sequenced on a MiSeq instrument (Illumina). The raw FASTQ files were submitted to NCBI, accession number GSE223216. (#2) RNA was extracted using the RNeasy Plus Mini Kit (Qiagen) with an additional mechanical lysis using MP Bio Fast Prep-24 in 1.5-ml screw cap tubes containing RNase-free acid-washed beads and RLT Plus Buffer (Qiagen). Samples were shaken at 4 m/s for 40 s and then put on ice for 60 s, repeated 4 times. Samples were centrifuged at top speed in a microfuge for 10 min to remove beads and cellular debris. The aqueous phase was transferred to gDNA eliminator column (RNeasy kit), following the manufacturer's instructions. RNA sample quality was tested using the Bioanalyzer High Sensitivity RNA Analysis Pico Kit (Agilent) and an Agilent 2100 Bioanalyzer. Samples were sent to Novogene (Sacramento, CA, USA) for cDNA synthesis and sequencing. The raw FASTQ files were submitted to NCBI, accession number GSE223217.

#### Reference genome

Parental directory C_albicans_SC5314 Assembly 22 found on CGD website (candidagenome.org; Skrzypek *et al.* 2017) was used as a reference genome for both RNA-seq experiments.

#### Bioinformatics analysis

Using Geneious software, version Geneious Prime, FASTQ files of the single-end reads were paired, trimmed using the BBDuk plug-in, and mapped to the reference genome. Raw reads from the 2 alleles were combined, and expression differences between the WT (OHWT) and mutant (TF021) were determined using the DESeq2 (v1.30.1) algorithm (Love *et al.* 2014) in R (v4.0.2). A cut-off of 10 reads total across the compared samples was the threshold for a gene to be included in the data set, and the apeglm algorithm was used as the common LFC shrinkage option to minimize variability of lowly expressed genes (Zhu *et al.* 2018). The *P*-values provided were adjusted with a Benjamini–Hochberg false discovery rate. Statistically significant differentially expressed genes had a *P*-value of 0.05 or less and a log2 fold change (up or down) of 0.58 or greater.

### qRT-PCR analysis

Quantitative real-time polymerase chain reaction (qRT-PCR) assays were performed on the same samples as for RNA-seq experiment (Ghosh *et al.* 2015). Cell collection and RNA extraction was performed as listed above. Primers used for *PHO100*, *FRE7*, *FRE30*, *FET31*, *FTR1*, *CFL2*, *OPT1*, and *TNA1* are listed in Table 2, and primers for *ADE13* and *NUP* and the reference gene *TEF1* were described (Wangsanut *et al.* 2017). The *P*-values for qRT-PCR were calculated using Student's *t*-test function in Excel. Pearson correlation coefficient (*r*) comparing relative fold changes obtained from RNA-Seq and qRT-PCR was calculated in Excel.

**Table 2.** Primers.

Name	Type	Sequence 5′ → 3′
*PHO100*	Forward Reverse	TCCAGGTGTTGCTTTCTCTG GCGTAAATGGTAGATGGTTCTTTG
*FRE7*	Forward Reverse	CTATCCCTAAGCCCAAATACTG CCATCACTTACCGTGGAATC
*FRE30*	Forward Reverse	CAAGGTCCCATTCAAATTCAC CCTAATAACGACGCACCATAG
*FET31*	Forward Reverse	GCCGGTGTCTTAGGTTTAG CATCAAAGTCGACATCCAAATC
*OPT3*	Forward Reverse	GTCCTTCTGGAGAGTAATTAAGG CATTCTGGAACTTCAGGGTAG
*TNA1* *FET34* *FET3* *OCT1* *CCC2* *PHO87* *VTC3*	Forward Reverse Forward Reverse Forward Reverse Forward Reverse Forward Reverse Forward Reverse Forward Reverse	GTGCTCCTTGGCCTATTTC CATTGATTTGCTCACGTTCTG AGAGACTTGGATGTGTACTTTG TTGTTTGAAGGGCTAGAAGAG CGAAGATCCTCAAGCCATTC TGTTGGCAGCAGCATTAC CCTTCTGTGTTGATGGAGTC CCAAGGAGACTTTCAGGTAAC TCTGGTACCGATATTGCTATTG TAGCGGTTGAGATTTGTAAGG CCCTGGGTTAAAGAAGTTGGAG GGGAATCCTGAAGTTGGTAATC GCAGAAGATCCAACCCATTAG CAATTTCCTCATCGGAATC

### Inductively coupled plasma mass spectrometry

Samples were prepared for ICP-MS analysis as described (Rosenfeld *et al.* 2010). Three cultures of each strain were grown overnight at 30°C in YPD and diluted to OD_600_ = 0.1 in 20 ml of YPD or YPD containing 13 mM CuSO_4_ (Fig. 7) or in 20 ml of SD + Arg with and without 10 mM potassium phosphate (Fig. 8), as described (Ikeh *et al.* 2016). Cultures were placed in a 30°C shaker and grown to OD_600_ of ∼1.0. A sample volume corresponding to 20 OD_600_ units was pelleted by centrifugation, washed 3 times with 10 mM Tris–HCl, pH 8.0, 1 mM EDTA, and pellets were digested overnight in 1 ml of 70% nitric acid at 95°. Cell debris was removed twice via centrifugation at 18,000 × *g*, and the clarified solution was diluted to 2% nitric acid using Milli-Q water. ICP-MS analysis was performed using an Agilent 7700 Series ICP-MS (Fig. 7) or an Agilent 7800 Series ICP-MS (Fig. 8) according to manufacturer specifications. The ICP-MS standards for calcium, cobalt, copper, iron, magnesium, manganese, phosphate, potassium, sodium, and zinc were purchased from Fluka Analytical and were diluted in 5% nitric acid. Three biological samples were each measured in 3 technical replicates. The counts per second output for the samples were converted to nmol/10^9^ cells after comparison with the standard curves. Averages from the biological and technical replicates were determined, and the standard deviations are presented in the graphs.

### Whole-genome sequencing and analysis

Strains were inoculated from single colonies into 5 ml of YPD medium and grown overnight at 30°C. Cells were pelleted in a microfuge for 10 min, resuspended in 180-*µ*l lysis buffer (1% SDS, 0.1 M NaCl, 10 mM Tris pH 8.0, 1 mM EDTA, and 2% Triton X-100), and transferred to a 1.5-ml screw cap tube containing ∼200 *µ*l of 0.1-*µ*m glass beads and 200 *µ*l of phenol:chloroform:isopropanol (25:24:1). Samples were shaken in a MP-Fast Prep-24 at 4 m/s for 25 s at 4°C; 200 *µ*l of 10 mM Tris–HCl, pH 8.0, 1 mM EDTA, was added; and samples were centrifuged for 10 min at top speed in a microfuge. The (top) aqueous layer was transferred to a new Eppendorf tube. Ethanol (1 ml of 100%) was added to the sample, mixed by inversion 10 times, and the DNA was pelleted for 10 min in a microfuge. The DNA pellet was resuspended in 400 *µ*l of 10 mM Tris–HCl, and pH 7.5, 1 mM EDTA. RNase was added to 7.5 *µ*g/ml, and samples were incubated for 45 min at 37°C. To precipitate the DNA, ammonium acetate was added to 100 mM, and ethanol was added to a final concentration of 70% prior to mixing. The samples were placed at −20°C for 30 min followed by centrifugation for 10 min. The pellets were washed in 1 ml of ice-cold 70% ethanol, and the DNA pellets were air-dried for 30 min. The pellets were each resuspended in 50 *µ*l of sterile, molecular biology grade water.

DNA samples (of 6 *µ*g or more) were sent to the SeqCenter (Pittsburgh, PA, USA) for whole-genome DNA sequencing using the Illumina 400 Mbp (2.67 M reads) Sequencing Package. The DNA sequencing was returned as fastq.gz files and also included the total reads, total read pairs, total base pair quality score, percent base pair coverage, and md5sum scores. Paired end reads were mapped in Y_MAP_ (Abbey *et al.* 2014) using reference genome SC5314 (var. A21-s02-m09-r07), and corrections, GC content bias and chromosome-end bias, were applied, as described (Selmecki *et al.* 2006, 2015).

## Results

### The *grf10*Δ mutant shows resistance to copper toxicity and exhibits a gene dosage effect

**Fig. 1.**
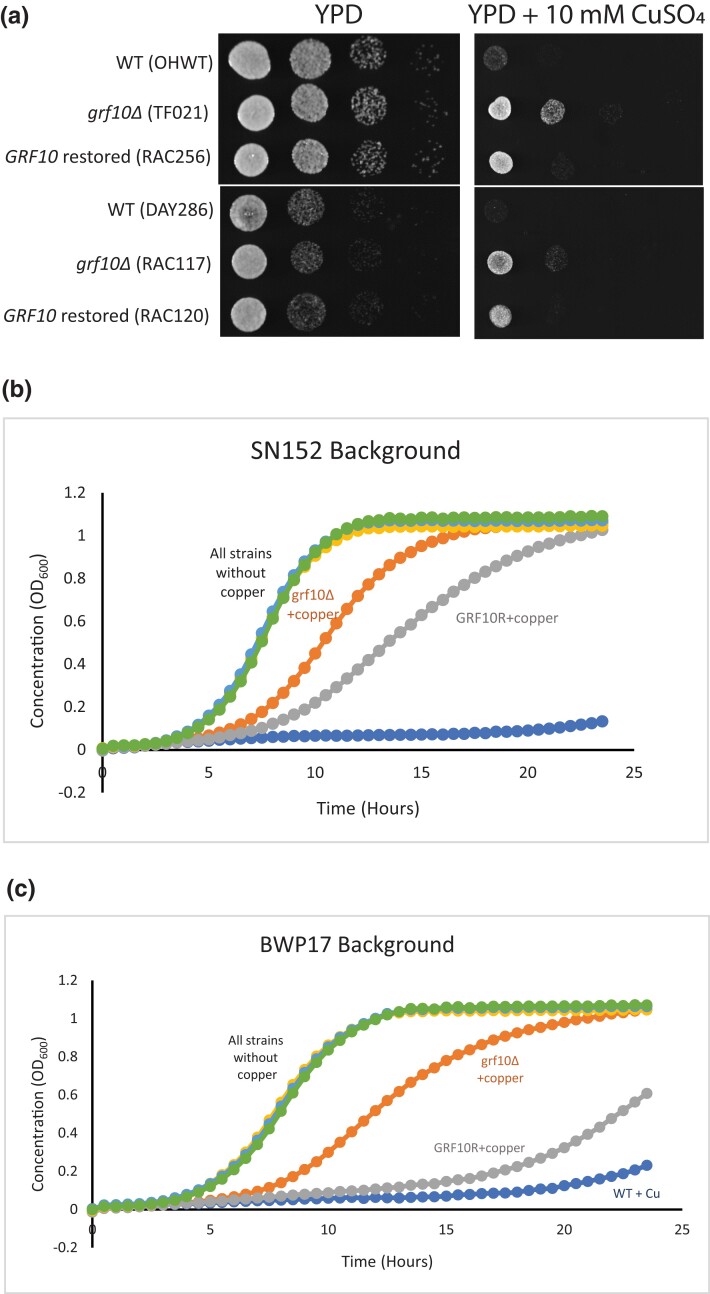
Loss of Grf10 confers resistance to copper in a gene dosage manner. a) Overnight cultures of wild-type strains (OHWT, DAY286), *grf10*Δ mutants (TF021, RAC117), and heterozygous-restored strains (RAC256, RAC120) were serially diluted and spotted on solid YPD medium with or without 10 mM copper and incubated at 30°C for 16 h. b and c) BWP17 and SN152 background growth (OD_600_) of the same cultures as in (a) was followed in YPD or YPD with 12 mM CuSO_4_ for 24 h. Graphs show the average OD_600_ reading from 3 biological replicates. See labels on graph; key: YPD: wild type in yellow; *grf10*Δ, light blue; restored, green; YPD + copper, wild type in dark blue; *grf10*Δ, orange; restored, gray.

Strains that lack the transcription factor Grf10 exhibit resistance to excess copper (10 mM CuSO_4_) (Homann *et al.* 2009). We note that these excess copper conditions represent toxic concentrations; nonetheless, we were interested in investigating this phenotype. We extended this observation by examining resistance in homozygous-null and heterozygous-restored strains (i.e. strains returned to heterozygosity by reintroducing 1 allele) in both the BWP17 and SN152 backgrounds (Fig. 1a). We confirmed that the *grf10*Δ mutant exhibits resistance as compared to the isogenic WT when presented with excess copper, and this phenotype is stronger in the BWP17 background than in SN152. To quantify the effect of copper on growth, we measured the doubling times of each strain growing in YPD liquid medium with or without the addition of 12 mM CuSO_4_ (Fig. 1b). The doubling times were the same for all of the strains grown in YPD (∼1.14 h); however, all of the strains grown in excess copper exhibited an increase in doubling time. The doubling time for the WT strain (DAY286) in the presence of high copper was 12.52 ± 2.02 h, growing significantly more slowly, and cell growth ceased, plateauing at an OD_600_ of ∼0.2. Strikingly, the *grf10*Δ mutant grew much better than the WT strain in the presence of copper and showed a gene dosage effect (Fig. 1b); the heterozygous-restored strain RAC120 doubled at ∼5 h (5.35 ± 2.67 h), and the null mutant RAC117 doubled ∼1.8 h (1.81 ± 0.09 h), which is only 1.6-times slower than when copper was not present. We found similar results with the strains in the SN152 background (Fig. 1c). These results suggest that the levels of Grf10 may be important for the expression of genes associated with copper uptake or sequestration.

### The metal resistance is specific to copper

**Fig. 2.**
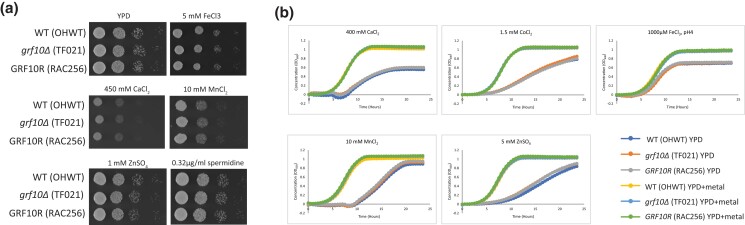
The *grf10*Δ mutant is not sensitive to other metals. a) Overnight cultures of the indicated strains (SN152 background) were serially diluted 1:10, spotted on YPD medium supplemented with the indicated metals, and incubated at 30°C for 18 h. b) Growth (OD_600_) of the same strains in YPD medium supplemented with 200 mM CaCl_2_, 1.5 mM CoCl_2_, 1 mM FeCl_3_ at pH 4, 10 mM MnCl_2_, and 4 mM ZnSO_4_ and followed for 24 h (additional concentrations tested are in Supplementary Figs. 1–5). Graphs show the average OD_600_ from 3 biological replicates. The key is the same as in Fig. 1 (legend is bottom right corner).

It is possible that the strong copper resistance phenotype of the *grf10*Δ mutant was due to a general defect in overall metal homeostasis. To test this, we assessed growth of the WT, *grf10*Δ mutant, and the heterozygous-restored strains on solid YPD medium supplemented with 450 mM calcium chloride, 5 mM iron chloride, 10 mM manganese chloride, 1 mM zinc sulfate, and 0.32 *µ*g/ml spermidine (Fig. 2a). Spermidine is an organic cation that was used to determine whether this resistance effect was due to overall positive charge, as opposed to being specific to metals (Ikeh *et al.* 2016). There was no difference in colony growth with any of these cations.

To test a broader range of concentrations and to ensure that we could detect growth differences, we assessed growth of the WT, *grf10*Δ mutant, and restored strains from both strain backgrounds in liquid medium supplemented with various metals. Figure 2b shows the growth curves for the SN152 background at 1 concentration for each metal tested; we supplemented YPD with 200 mM CaCl_2_, 1.5 mM CoCl_2_, 1 mM FeCl_3_ (at pH 4), 12 mM MnCl_2_, and 4 mM ZnSO_4_. The growth curves for the full range of concentrations tested and in both strains are found in Supplementary Figs. 1–5. All strains grew worse in the presence of the tested metal than in its absence, with slower doubling times and reaching stationary phase at a lower OD_600_. Importantly, all 3 strains responded in the same way to each addition, indicating that there was no general defect in metal homeostasis. Thus, the phenotype is specific to copper, suggesting that Grf10 is involved in expression of genes important for copper metabolism.

### Copper toxicity response is dependent on Grf10 conserved residues D302 and E305

Grf10 contains a conserved interaction region, located from amino acids 270–353, that is important for it to activate expression, likely due to a co-regulator interaction (Wangsanut *et al.* 2017; Wangsanut *et al.* 2018). The *grf10*-D302A mutant is completely defective in adenine regulation and in the response to filamentation, and the *grf10*-E305A mutant is partially defective for filamentation and is normal for adenine regulation (Wangsanut *et al.* 2018); neither mutation affects protein stability. We examined whether conserved residues D302 and E305 are important for the copper sensitivity of the *GRF10* strain.

**Fig. 3.**
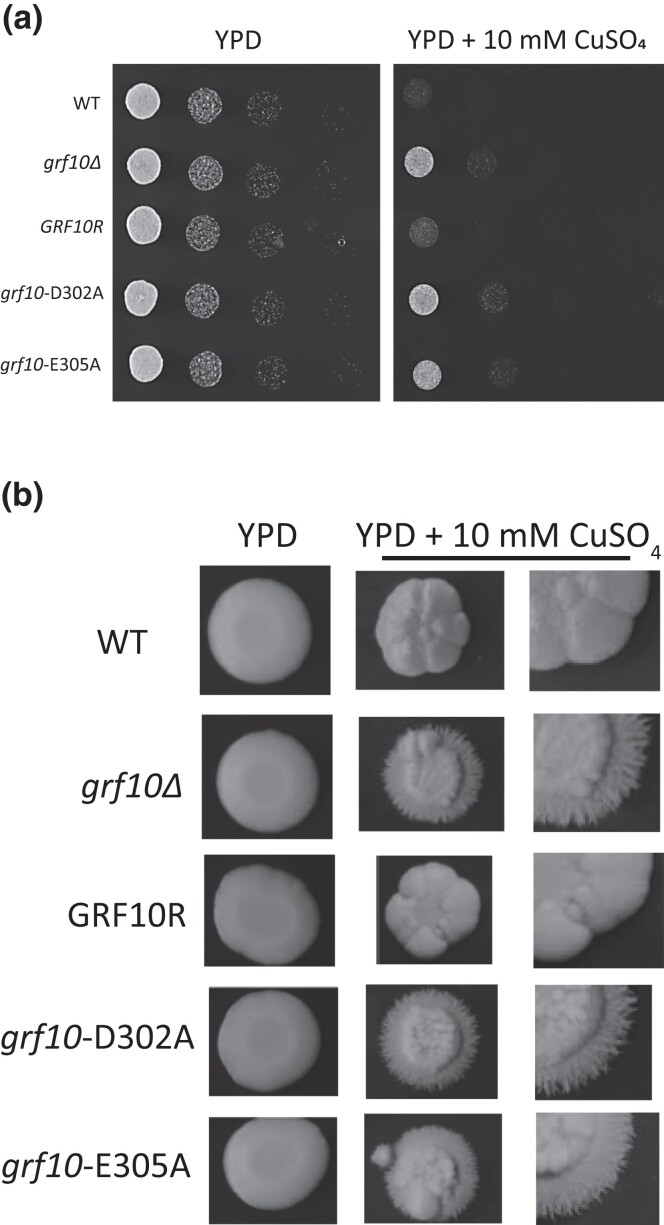
Amino acids D302 and E305 of Grf10 are critical for copper sensitivity and morphology. a) Overnight cultures of the WT strain (DAY286), *grf10*Δ mutant (RAC117), heterozygous-restored strains expressing *GRF10* (RAC120), or *GRF10* with substitution mutations, *grf10*-D302A (RAC259) and *grf10*-E305A (RAC260), were spotted onto YPD with or without 10 mM CuSO_4_ and grown as in Fig. 1. b) The same plates from (a) were cultured for 7 days at 30°C and photos were taken of a single whole colony or edges of a colony.

To test this, we used previously constructed strains, *grf10*-D302A or *grf10*-E305A, which contain 1 allele of *GRF10* with either the D302A or E305A point mutation. We grew these strains, the isogenic WT, the *grf10*Δ mutant, and the restored strain *GRF10R* on solid YPD medium with or without 10 mM copper sulfate (Fig. 3a). We found that both of the mutant *grf10* alleles completely failed to reverse the copper sensitivity; in other words, the substitutions behaved as a nonfunctioning null allele. This result indicated that regulation of copper toxicity is dependent on both D302 and E305 of Grf10 and suggested that these residues mediate the interaction between Grf10 and a specific protein partner in response to excess copper.

We observed that with prolonged incubation on YPD containing copper sulfate (30°C for 7 days), peripheral hyphae were produced from the colonies that expressed the defective alleles of *GRF10—*the *grf10*Δ, *grf10*-D302, and *grf10*-E305—whereas the WT counterpart and the heterozygous-restored strains did not form hyphae (Fig. 3b). This intriguing result suggests that Grf10 normally inhibits hyphal formation when the cell encounters toxic levels of copper.

### Evolution of WT demonstrates copper resistance when grown in excess copper

The WT colony on YPD + 10 mM CuSO_4_ at 7 days of incubation had an irregular colony morphology as compared to growth on YPD (Fig. 3b). We observed papillae growing above the background at earlier times (see Fig. 3a and Supplementary Fig. 6). Furthermore, we noted that the WT strain had late growth in liquid YPD + CuSO_4_ medium (Fig. 1b and c). We hypothesized that the papilla were individual yeast cells that had mutated under selection to resist the high levels of copper; those colonies continued to grow and eventually merged over the week to become the lumpy colony (Fig. 3b). If so, the trait of growth in high copper should be stable due to these mutations.

**Fig. 4.**
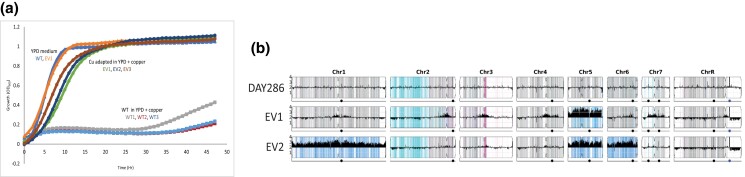
Evolution of WT demonstrates copper resistance when grown in excess copper and contains chromosomal abnormalities. a) Overnight cultures of the WT strain (DAY286) and putatively force evolved papilla (EV; originating from DAY286) were grown overnight in 5 ml of YPD, normalized to OD_600_ of 0.2, and grown in YPD or YPD with 12 mM CuSO_4_. Three biological replicates of each WT (squares of gray, blue and red) and EV (circles of brown, navy and green) strains were used for the YPD plus copper addition, and only 1 biological replicate was used for the WT (blue triangle) and EV (orange triangle) strains for the YPD control. b) YMAP plots illustrating genome-wide changes occurring in EV1 and EV2 strains as compared to the parental WT (DAY286) strain. The gray lines indicate equal representation of both alleles (A and B) of each gene across the chromosome, and the aqua and pink indicate previously described loss-of-heterozygosity events on chromsomes 2 and 3. In the evolved strains (EV1, EV2), the blue regions and allele counts above the diploid levels of 2 indicate aneuploidy (trisomy and tetrasomy) within the population.

To test this, we selected several papillae from the growth of the WT strain on YPD + 12 mM CuSO_4_ and grew them on solid YPD medium—i.e. growth conditions without high copper selection. Three of these “evolved” strains and 3 colonies from the original (nonselected) WT strain were used to inoculate cultures with and without high copper, and growth was measured in liquid culture over time (Fig. 4a). We found that the EV strains (EV1-3) were able to grow immediately, whereas the WT cultures grown in the presence of excess copper exhibited a growth delay, similarly to what was seen in Fig. 1.

The most likely explanation for the EV phenotype is aneuploidy with increased copy number of genes important to the copper selection (Berman 2016). To examine this, we performed whole-genome sequencing on the parental WT (DAY286) and 2 EV strains, EV1 and EV2. As shown in Fig. 4b, the EV strains EV1 and EV2 showed increased aneuploidy of chromosome 5; EV2 also exhibited aneuploidy of chromosomes 1 and 6. Chromosome 5 is 1.19 kb long and carries 523 ORFs (Skrzypek *et al.* 2017); 2 of the genes located on chromosome 5 and related to copper homeostasis are *CUP1* that encodes the copper metallothionein and *CCC2* that encodes a metallochaperone. These genes may have driven selection for increased copy number.

### Identification of global *GRF10*-dependent genes

To gain insights into misregulated genes involved in copper metabolism, we analyzed an RNA-seq data set comparing differential gene expression from the *grf10*Δ mutant (TF021) and isogenic WT (OHWT) strains grown in YPD medium for 1 and 4 h. Grf10-dependent genes were identified as those in which the expression was altered by at least 1.5-fold (log2 fold change = 0.58) and an adjusted *P-*value of 0.05 or lower (Tables 3 and 4; the complete data sets are found in Supplementary Tables 1 and 2).

**Table 3.** Grf10-dependent genes at 1 h.

Down or up in *grf10*Δ mutant	Gene	Locus	Average Log2 change	Classification and comments
Decreased	*GRF10*	C5_05080W	−3.775	Deleted Gene
	*PGA45*	C1_05960W	−1.779	
	*OPT1*	CR_02020C	−1.363	
	*GSY1*	CR_00780C	−1.354	
	*UCF1*	C2_08290C	−1.314	
	*IRO1*	C3_01360C	−1.271	P/Cu/Fe
	*HIS1*	C5_05320C	−1.259	marker
	*ALD5*	C2_02970C	−1.211	
	*GLG21*	C1_01360C	−1.168	
	*FRE7*	CR_07290W	−1.044	P/Cu/Fe
	*PGA10*	C4_00450C	−1.010	
	*FRE30*	CR_07280W	−0.955	P/Cu/Fe
	*FAS2*	C3_04830C	−0.941	
	*PHO87*	C1_05940W	−0.939	P/Cu/Fe
	*VTC4*	C4_03360C	−0.937	P/Cu/Fe
	orf10.1691	C3_01540W	−0.906	
	*GLK1*	CR_07150W	−0.897	
	*FET31*	C6_00480C	−0.853	P/Cu/Fe
	*CAT1*	C1_06810W	−0.851	
	orf9.4450.1	C1_07160C	−0.824	
	*ATO2*	C3_00930W	−0.798	
	*OSM2*	C1_13670W	−0.791	
	*TPI1*	C3_07440W	−0.782	
	*CTR1*	C6_00790C	−0.766	P/Cu/Fe
	*URA3*	C3_01350C	−0.741	
	*PGA53*	C4_01360W	−0.727	
	*PHO86*	C2_07820C	−0.716	P/Cu/Fe
	*PGK1*	C6_07820C	−0.714	
	*VTC3*	CR_03610C	−0.65	P/Cu/Fe
	*XYL2*	CR_10840C	−0.706	
	orf19.3053	C1_03510C	−0.696	
	*PHO84*	C1_11480W	−0.691	P/Cu/Fe
	*FAS1*	C5_00190C	−0.689	
	orf19.1964	C5_01070C	−0.635	
	*TFS1*	C5_00930C	−0.630	
	*CHT2*	C5_04130C	−0.621	
	*GPH1*	C7_00930W	−0.618	
	orf19.4246	C5_02380W	−0.611	
	*MAE1*	C6_01670W	−0.604	
	*OLE1*	C1_08360C	−0.604	
	*HEM13*	C3_04060C	−0.596	
	*ACC1*	CR_00640W	−0.595	
	*PGA13*	CR_08510W	−0.594	
	*PSA2*	C1_13160W	−0.594	
	*PIR1*	C2_08870C	−0.591	
Increased	*LEU2*	C7_00400W	2.670	marker
	*CRZ2*	CR_07060C	0.986	
	*ZCF21*	C4_00760W	0.953	
	*HGT8*	C2_01010W	0.888	
	orf19.4875	C1_10080W	0.766	
	orf19.4643	C4_01420W	0.731	
	orf19.1975	C5_00920W	0.701	
	orf19.1473	C2_01630W	0.683	
	*EBP1*	C6_01180C	0.617	
	*ALS4*	C6_04130C	0.614	

**Table 4.** Grf10-dependent genes at 4 h.

Down or up in *grf10*Δ	Gene	Locus	Average Log2 change	Classification and comments
Decreased	*HGT17*	C4_01070W	−5.027	
	*PHO100*	C1_07430W	−4.653	P/Cu/Fe
	*GRF10*	C5_05080W	−3.708	Deleted Gene
	*CTN1*	C1_01740W	−3.271	
	*TNA1*	C5_03060C	−3.261	
	*GAP2*	C3_05580C	−2.922	
	*UCF1*	C2_08290C	−2.910	
	*HPD1*	C6_02890C	−2.829	
	*PXP2*	C3_01930W	−2.631	
	*ADH2*	C1_08330C	−2.631	
	*GIT1*	C2_06590C	−2.241	P/Cu/Fe
	*FDH1*	CR_05170C	−2.324	
	*ALD6*	C4_05130C	−2.282	
	*GIT4*	C5_00870C	−2.243	
	*HGT12*	C7_00280W	−2.157	
	orf19.7279.1	CR_08830W	−2.092	
	*ICL1*	C1_04500W	−1.981	
	*HIS1*	C5_05320C	−1.935	Marker
	*BTA1*	C1_11490C	−1.878	
	*HGT2*	C1_02110C	−1.854	
	*AMO1*	C2_03120W	−1.654	
	*BLP1*	C1_12850W	−1.610	
	*CYB2*	C1_13630W	−1.593	
	*NUP*	C7_01560C	−1.556	ADE/1 carbon
	*FET31*	C6_00480C	−1.525	P/Cu/Fe
	*OPT3*	CR_02220C	−1.495	
	*OPT1*	CR_02020C	−1.368	
	*IRO1*	C3_01360C	−1.310	P/Cu/Fe
	*PGA13*	CR_08510W	−1.258	
	orf19.4450.1	C1_07160C	−1.150	
	*PHO87*	C1_05940W	−1.120	P/Cu/Fe
	*DUR1,2*	C1_04660W	−1.074	
	*STF2*	C1_00250W	−1.053	
	*SNZ1*	C1_02590C	−1.037	
	*FTR1*	C1_14130W	−1.034	P/Cu/Fe
	*CAT8*	C1_08190C	−0.959	
	*AIP2*	C3_03040W	−0.938	
	*CFL2*	C4_05780C	−0.935	P/Cu/Fe
	*COI1*	C1_07900W	−0.926	
	*URA3*	C3_01350C	−0.920	
	*CAN2*	C6_01060C	−0.893	
	*VTC4*	C4_03360C	−0.836	P/Cu/Fe
	*GDH3*	C4_06120W	−0.836	
	*FRE10*	C4_04320W	−0.809	P/Cu/Fe
	*ADE13*	CR_06150C	−0.807	ADE/1 carbon
	*PHO84*	C1_11480W	−0.802	P/Cu/Fe
	orf19.1461	C2_01540W	−0.787	
	*POT1-2*	C2_00780W	−0.748	
	*SEO1*	CR_06660W	−0.727	
	*CHT2*	C5_04130C	−0.712	
	*CAN1*	C6_00960W	−0.689	
	*ARG5,6*	C1_09290C	−0.684	
	*AMO2*	C2_06700W	−0.661	
	orf19.4330	C5_03030W	−0.648	
	orf19.4550	C1_01750W	−0.644	
	*POT1*	CR_00150C	−0.641	
	*DUR3*	C5_03480C	−0.633	
	*VTC3*	CR_03610C	−0.629	P/Cu/Fe
	*MET13*	C3_02950C	−0.628	
	*MTD1*	C4_04720W	−0.619	ADE/1 carbon
	*CWH8*	C1_02250W	−0.610	
	*MEP2*	C4_00430W	−0.580	
Increased	*ASR2*	CR_08890C	1.589	
	*WH11*	C2_05180W	1.293	
	*SOU1*	C4_06390W	1.273	
	*LEU2*	C7_00400W	1.175	Marker
	orf19.4216	C5_02110W	0.925	
	*GLX3*	C3_02610C	0.933	
	*HSP12*	C5_02080C	0.925	P/Cu/Fe
	*TLO10*	C4_07250C	0.921	
	*CSP37*	CR_01470W	0.907	
	*HSP70*	C1_13480W	0.795	
	orf19.6113	C1_00030C	0.794	
	orf19.675	C1_11270W	0.787	
	*RTA3*	C2_06460W	0.760	
	orf19.5364	C2_10810W	0.721	
	*ASR3*	C2_03790C	0.712	
	*HGT8*	C2_01010W	0.710	
	*CRD2*	C4_01160W	0.703	P/Cu/Fe
	*IEF1*	C1_04750W	0.683	
	*PAD1*	C6_03630W	0.668	
	*PRE1*	C5_05310W	0.628	
	*CSH1*	C1_04020C	0.627	
	orf19.6077	C1_00310W	0.615	
	orf19.6184	C3_07880C	0.609	

**Fig. 5.**
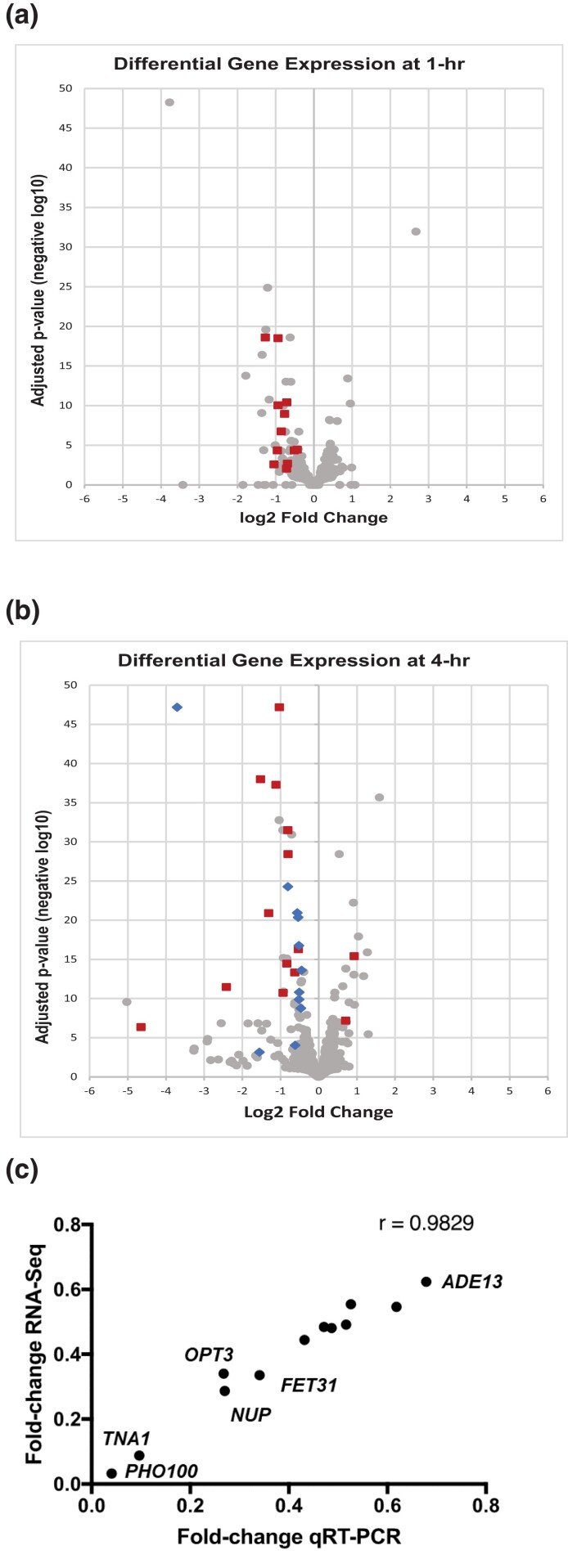
Differential gene expression in the *grf10*Δ mutant. Volcano plots (Log2 differential expression vs. absolute value of the confidence score) showing both alleles of the genes. Differentially expressed genes were defined as those with a confidence score >6. Alleles of the genes associated with processes discussed in the text are denoted as red squares for copper, iron, and phosphate metabolism; blue diamonds for purine nucleotide and 1-carbon metabolism; and gray circles for the remainder. a) 1-h post-inoculation. b) 4-h post-inoculation. Note, *HIS1* is not shown (point at −1.9, 101). c) Correlation between RNA-seq and qRT-PCR experiments.

As expected, we saw a significant difference in expression for *GRF10* and for marker genes *LEU2* and *HIS1* integrated at *GRF10* rather than their native loci. Of the remaining genes, 52 genes showed differential expression in the *grf10*Δ mutant at 1 h following inoculation (43 genes with decreased expression and 9 genes with increased expression), and 82 showed differential expression in the *grf10*Δ mutant at 4 h (60 genes with decreased and 22 genes with increased expression); see volcano plots in Fig. 5a and 5b and GO term analyses in Supplementary Tables 3 and 4. Twelve of these genes were in common between the 2 time points, and half of these are involved in phosphate metabolism and ion transport (*FET31*, *IRO1*, *PHO84*, *PHO87*, *VTC3*, and *VTC4*). Finally, we verified the expression data using qRT-PCR for 10 genes: *PHO100*, *FRE7*, *FRE30*, *FET31*, *FTR1*, *CFL2*, *OPT1*, *TNA1*, *ADE13*, and *NUP* (Fig. 5c); expression differences were correlated between the 2 methods (Pearson correlation coefficient = 0.983).

Ten genes associated with phosphate, iron, and copper homeostasis (*PHO84*, *PHO86*, *PHO87*, *VTC3*, *VTC4*, *IRO1*, *FRE7*, *FRE30*, *FET31*, and *CTR1*) are differentially expressed at 1-h post-inoculation (red squares in Fig. 5a). Prominent among the differentially regulated genes at 4 h are those involved in iron, copper, and phosphate metabolism (red squares in Fig. 5b): 11 genes with lower expression included *PHO100*, *GIT1*, *PHO84*, *PHO87*, *VTC3*, *VTC4*, *IRO1*, *FTR1*, *FET31*, *FRE10*, and *CFL2* and 2 with increased expression *CRD2* and *HSP12*. Additionally, we see differential expression of genes involved in adenylate and 1-carbon metabolism (blue diamonds in Fig. 5b), as previously described (Wangsanut *et al.* 2017). These findings suggest that Grf10 is involved, directly or indirectly, with phosphate, iron, and copper homeostasis.

### Gene expression in the *grf10*Δ strain shows a largely WT response to a copper challenge

To investigate the responses to copper in the *grf10*Δ mutant and WT strains, we examined differential gene expression after challenging cells with 12 mM copper sulfate using RNA-seq. The response to copper altered the expression of 1,283 in WT cells (adjusted *P-*value < 0.05 and log2 fold change of >0.58) and 1,164 in the *grf10*Δ mutant (Supplementary Tables 5 and 6, respectively). There was no significant difference in the expression of *GFR10* in WT cells upon copper treatment (Supplementary Table 5).

**Fig. 6.**
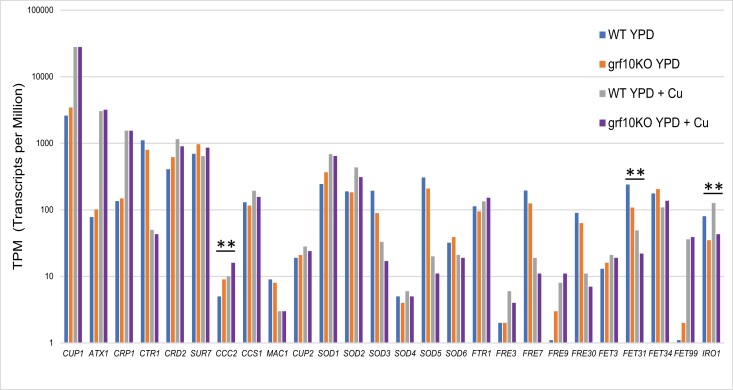
Differential gene expression of copper and iron metabolic genes in the *grf10Δ* mutant in response to high copper. The transcriptional responses of the WT and *grf10*Δ strains to growth on YPD (blue and orange bars, respectively) or YPD + 12 mM CuSO_4_ (grey and purple bars, respectively). RNA was isolated from 3 cultures for each strain/growth condition. The average TPM values from 3 replicates for the genes are shown on a log scale (see Supplementary Tables 5 and 6).

Given the copper resistance phenotype, we looked closely at the expression of genes known to be involved in copper, iron, and superoxide metabolism (Askwith *et al.* 1994; Morrissey *et al.* 1996; Chibana *et al.* 2005; Douglas *et al.* 2011; Cheng *et al.* 2013; Garcia-Santamarina and Thiele 2015; Li *et al.* 2015; Smith *et al.* 2017; Gerwien *et al.* 2018; Khemiri *et al.* 2020). The *grf10*Δ mutant strain responded to the copper challenge nearly the same as the WT strain (Fig. 6), upregulating key survival genes such as the *CUP1* metallothionein, the *ATX1* copper metallochaperone, the *CRP1* copper extrusion pump, and the *SOD1* superoxide dismutase, as well as repressing the copper transporter *CTR1.* Three genes showed significant differential expression in the 2 strains in high copper: the iron utilization gene *IRO1* was expressed at 36% of the WT, the multicopper oxidase gene *FET31* was expressed at about half the level of the WT, and the intracellular copper transporter gene *CCC2* was expressed 1.6-fold higher. All of the genes showing differential expression in the *grf10*Δ mutant in high copper are listed in Supplementary Table 7).

Beyond the genes noted above, 224 genes are misregulated in the *grf10*Δ mutant under high copper conditions (110 genes have increased expression and 114 with decreased expression) (Supplementary Table 7). Not surprisingly, genes with lower expression included those involved in adenylate and 1-carbon metabolism as well as 2 genes (*SNZ1* and *SNO1*) involved in pyridoxal phosphate biosynthesis. The gene showing the greatest increase in expression in the mutant was *CHA1*, which encodes a Ser/Thr dehydratase and is induced in low iron (Lan *et al.* 2004).

### ICP-MS reveals similar intracellular copper accumulation in mutant and WT cells

**Fig. 7.**
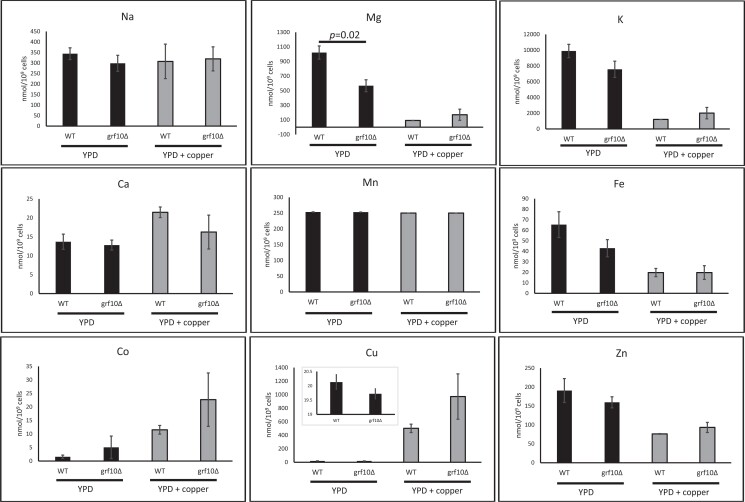
WT and *grf10*Δ strains accumulate copper and other metals to the same extent. WT (DAY185) and *grf10*Δ (RAC117) strains were grown to mid-log phase in YPD with and without 13 mM CuSO_4_ and prepared as described in *Materials and Methods*. Samples were analyzed via ICP-MS, solid black bars indicate samples from YPD-grown cells, and gray bars indicate samples from cells grown with CuSO_4_. The iron results are the sum of isotopes Fe-54 and Fe-57; inset in the copper panel is the expansion showing the values between 0 and 20 mmol per billion cells. Results for each element are shown as averages from 3 independent cultures and 3 technical replicates, and the error bars indicate standard deviations. Statistical analysis was conducted using Student's *t*-test for comparisons between WT and *grf10*Δ strains, and the significant *P-*value is indicated above the graph.

Given the difference in expression of copper and iron transporters (Fig. 5) as well as the growth resistance to copper (Fig. 1), we asked whether the *grf10*Δ mutant strain would accumulate lower levels of copper. We determined the intracellular metal accumulation using ICP-MS. We assessed the levels of copper and 8 additional elements—calcium, cobalt, iron, magnesium, manganese, potassium, sodium, and zinc (Rosenfeld *et al.* 2010) (Fig. 7). Under normal YPD culturing conditions, we found no difference in the intracellular copper accumulation in the *grf10*Δ mutant and WT strains, with both strains accumulating ∼20 nmol per billion cells. Magnesium accumulated to 57% of the WT levels in the *grf10*Δ mutant under normal growth conditions (*P* = 0.02). There were no changes in the steady-state levels of the other metals examined (Fig. 7).

When cells were grown in YPD with 13 mM copper sulfate added, the WT and *grf10*Δ mutant strains had no significant difference in copper accumulation (*P* = 0.08). Cobalt levels increased in a pattern that was similar to that of copper, higher in the presence of copper in both strains (*P ≤* 0.02), but not significantly different between mutant and WT (*P =* 0.08). We detected lower levels of iron, magnesium, potassium, and zinc (*P ≤* 0.02) in both the mutant and WT strains (Fig. 7). Given this, we conclude that the WT and the *grf10*Δ mutant do not differ in their accumulation of intracellular metals. Furthermore, these results indicate that the *grf10*Δ mutant and WT cells accumulate the same amount of copper in spite of its copper toxicity resistance phenotype.

### The *grf10*Δ mutant accumulates lower levels of phosphate

**Fig. 8.**
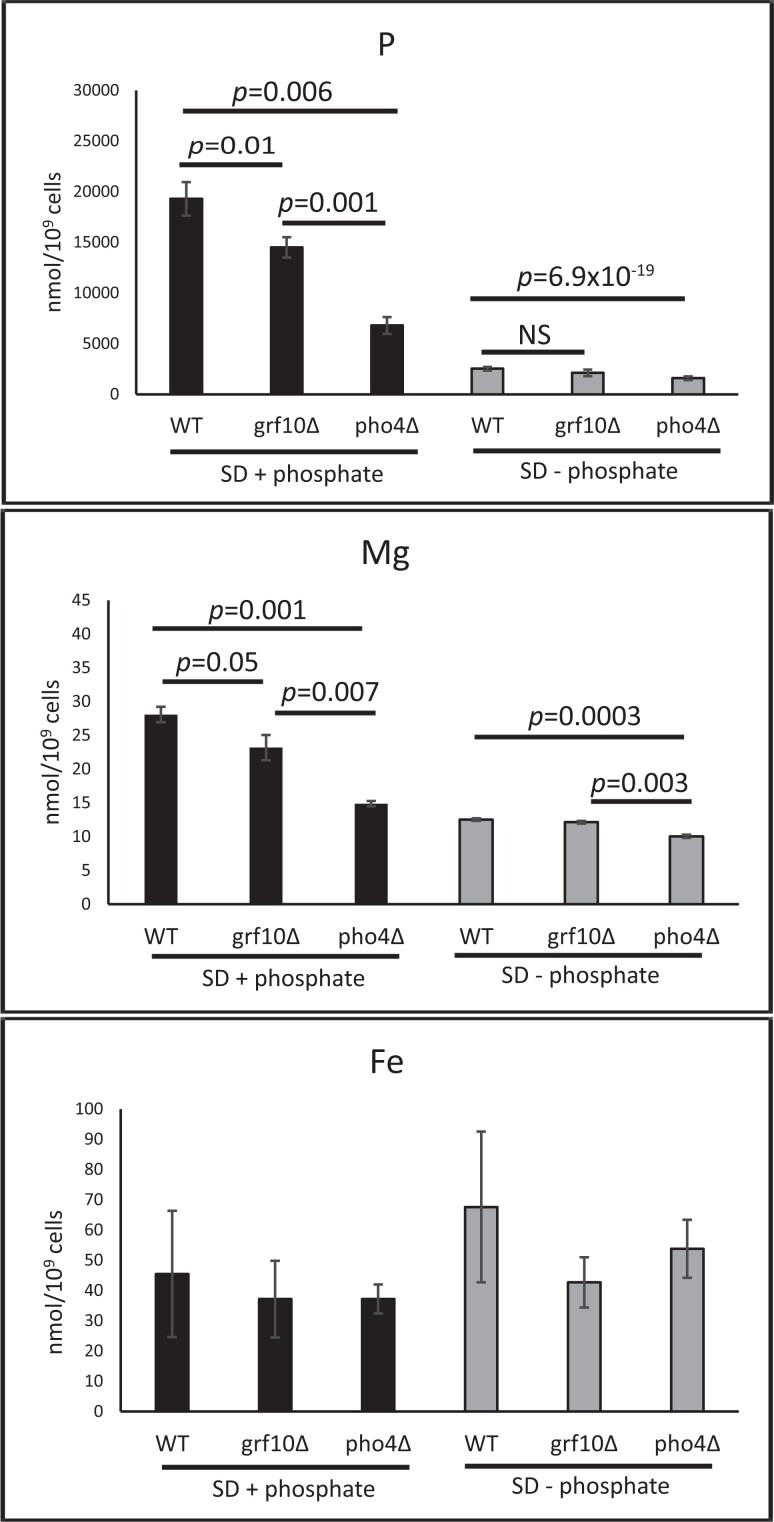
The *grf10*Δ and *pho4*Δ mutants show differences in the intracellular accumulation of phosphorus and magnesium. WT (OHWT; derived from SN152), *grf10*Δ (TF021), and *pho4*Δ (JC1928; isogenic WT is SN152) strains were grown in SD + arginine or SD + arginine supplemented with 10 mM phosphate. Samples were prepared for ICP-MS as described in Fig. 7. Solid black bars indicate samples from phosphate-supplemented medium, and gray bars indicate samples lacking phosphate. Bars represent the averages from 3 independent cultures and 3 technical replicates, and the error bars indicate standard deviations. Statistical analysis was conducted using Student's *t*-test for comparisons between WT, *grf10*Δ, and *pho4*Δ strains, and significant *P*-values are indicated above the graphs. These data were collected using a different set of samples and different ICP-MS instrument from the data found in Fig. 7.

The identification that many genes in the *PHO* regulon showed lower expression in the *grf10*Δ mutant led us to hypothesize that phosphate metabolism is affected. We measured differences in phosphate accumulation using ICP-MS in the WT, *grf10*Δ and *pho4*Δ strains when cells were cultured in phosphate replete (10 mM potassium phosphate) and limiting (no addition) media. In addition to phosphorus, we examined accumulation of magnesium and iron (see Fig. 8, and Rosenfeld *et al.* 2010; Ikeh *et al.* 2016). The *grf10*Δ mutant accumulated ∼75% of phosphorus relative to the WT (*P* = 0.03) when the growth medium was supplemented with phosphate (Fig. 8). The *pho4*Δ mutant accumulated only about 30% of the WT levels of phosphorus (*P* = 0.006) under these conditions. When limited for phosphate, all 3 strains showed lower accumulation; only the *pho4*Δ was significantly different from the WT (*P =* 6.9 × 10^−19^). Magnesium, the typical counterion to phosphate, also showed a similar pattern as phosphate. The *grf10* strain accumulated magnesium to 82% of the WT (*P* = 0.05), and the *pho4* strain accumulated it to 54% of WT (*P* = 0.001).

We found no significant difference in the accumulation of iron between any of the 3 strains (Fig. 8). These results of lower phosphorus accumulation in the *grf10*Δ mutant are consistent with the decrease in *PHO* and *VTC* gene expression. Given this, it is possible that Grf10 and Pho4 transcription factors interact to regulate *PHO* genes.

## Discussion

Grf10 is a homeodomain transcription that plays roles in developmental processes such as morphogenesis, filamentation, biofilm formation, and white-opaque switching as well as in biosynthetic pathways such as adenylate synthesis and 1-carbon metabolism (Ghosh *et al.* 2015; Wangsanut *et al.* 2017, 2018; Rodriguez *et al.* 2020; Qasim *et al.* 2021). In this study, we examined the copper resistance phenotype, first described by Homann *et al.* (2009). We extended the initial observation, examined metal accumulation by ICP-MS, and identified novel Grf10-dependent genes involved in the uptake, sequestration, and storage of copper, iron, and phosphate. Resistance was restricted to high levels of copper, and it did not extend to other metals such as iron, calcium, manganese, or zinc, leading us to focus on copper uptake and/or sequestration as a mechanism for resistance. The *grf10*Δ mutant did not accumulate less copper than the WT strain, suggesting that the bioavailability of copper is different between the strains. The copper specific toxicity response was dependent on conserved residues D302 and E305 of Grf10 (Wangsanut *et al.* 2018).

The Grf10 residues D302 and E305 are in protein interaction region that has been conserved through fungal diversification (Wangsanut *et al.* 2018). In *Saccharomyces cerevisiae*, this region of *Sc*Pho2 is necessary for interactions with 3 co-regulators: *Sc*Pho4 for regulation of phosphate uptake and utilization, *Sc*Bas1 for regulation of adenylate and 1-carbon metabolism, and *Sc*Siw5 for mating type switching (Vogel *et al.* 1989; Daignan-Fornier and Fink 1992; Brazas *et al.* 1995; Barbaric *et al.* 1996; Magbanua *et al.* 1997; Zhang *et al.* 1997; Pinson *et al.* 2000; Bhoite *et al.* 2002; Hannum *et al.* 2002; Som *et al.* 2005). This same region is required for Grf10 to control the response to copper as well as filamentation and adenine prototrophy (Fig. 3 and Wangsanut *et al.* 2018). While the D302A mutation was defective in all phenotypes tested, the E305A mutation exhibited variable phenotypes—completely defective for copper resistance, partially defective for filamentation, and normal for ADE prototrophy—consistent with a model that Grf10 regulates these phenotypes with different co-regulators (Wangsanut *et al.* 2017, 2018). We hypothesize that Grf10 forms a ternary complex consisting of Grf10, a protein partner, and DNA to regulate genes involved in multiple cellular processes including adenine biosynthesis and 1-carbon metabolism, filamentation, and metal homeostasis.

Mac1 and Cup2 are the characterized transcription factors that regulate copper responsive genes during copper depletion and copper excess, respectively (Marvin *et al.* 2004; Schwartz *et al.* 2013; Smith *et al.* 2017). Grf10 likely works separately from Mac1 and Cup2 because deletions of each these genes show distinct copper phenotypes. We did not see any changes in the levels of *MAC1* or *CUP2* transcripts comparing the WT and *grf10Δ* mutant. Unlike the *grf10*Δ mutant that exhibits a resistance to excess copper, the *mac1*Δ mutant shows growth sensitivity under copper limitation, and the *cup2*Δ mutant shows growth sensitivity under copper excess (Homann *et al.* 2009). An extensive phenotypic analysis of transcription factor mutants revealed that several transcription factors conferred a similar copper resistance phenotype to that seen in the *grf10*Δ mutant: *rlm1*Δ, *crz2*Δ, *pho4*Δ, *cph2*Δ, *wor2*Δ, *orf19.2961*Δ, *isw2*Δ, *hap31*Δ, *efg1*Δ, *hap2*Δ, and *hap5*Δ strains (Homann *et al.* 2009). Grf10 could interact with 1 or more of these transcription factors to regulate the copper response.

Grf10 may play an important role in linking the response to copper with the hyphal developmental program by working downstream of or with Efg1 in Ras/cAMP/PKA pathway. The G-protein subunit Gpa2, cAMP, Efg1, and Grf10 are each required for both filamentation and the copper toxicity response (this study and Maidan *et al.* 2005; Homann *et al.* 2009; Schwartz *et al.* 2013; Ghosh *et al.* 2015; Azadmanesh *et al.* 2017). Likewise, Marvin *et al.* (2003) demonstrated that perturbation of copper acquisition due to mutations in the copper transporter gene *CTR1* promoted filamentation in *C. albicans*, consistent with our observation that filaments were produced in the *grf10*Δ mutant after prolonged incubation with excess copper. Importantly, Efg1 induces both *GRF10* expression during biofilm formation, possibly also during hyphal formation, and expression of copper and iron uptake genes (Stichternoth and Ernst 2009; Nobile *et al.* 2012; Rodriguez *et al.* 2020). Thus, Grf10 may work with Efg1 through the Ras/cAMP/PKA pathway to coordinate a copper response with hyphal growth.

### Grf10 may be involved in phosphate regulation

The identification of misregulation of genes involved in phosphate uptake and polyphosphate formation was unexpected. Several groups have reported that the *grf10*Δ mutant has no growth phenotype on media with limiting phosphate (Homann *et al.* 2009; Kerwin and Wykoff 2009; Wangsanut *et al.* 2017). Although *PHO* gene expression in *S. cerevisiae* requires both *Sc*Pho4 and *Sc*Pho2, expression of *PHO* genes in *Candida glabrata* requires only *Cg*Pho4; therefore, as the ascomycetes diverged, the PHO pathway was rewired, and *S. cerevisiae* has the novel dependence on a second transcription factor (Kerwin and Wykoff 2009). Thus, the dependence on Grf10 for expression of *PHO* genes in *C. albicans* was unexpected.

On the other hand, there are connections between phosphate metabolism and metal homeostasis. We and others found that both the *pho4*Δ and *grf10*Δ mutants show copper resistance (Homann *et al.* 2009; Ikeh *et al.* 2016). In *C. albicans*, *C. glabrata*, and *Cryptococcus neoformans*, the transcription factor Pho4 regulates the *PHO* regulon during phosphate limitation, and genes involved with nutrient transporters and metal homeostasis under phosphate replete conditions (Toh-e *et al.* 2015; Ikeh *et al.* 2016; He *et al.* 2017; Lev and Djordjevic 2018). It has been suggested that an expanded set of Pho4 targets, beyond the *PHO* regulon, could have evolved from a reduced requirement for the co-activator Grf10/Pho2 (Kerwin and Wykoff 2009; He *et al.* 2017). Thus, our results highlight the need to reconsider the relationship between Grf10 and Pho4 in *C. albicans*.

### Summary

We have expanded the understanding of Grf10-dependent target genes to multiple pathways: purine biosynthesis and uptake, yeast to hyphae switching, copper and iron response, and uptake of inorganic phosphate. Many genes in these pathways are required for virulence in murine models of *C. albicans* infection (Ramanan and Wang 2000; Donovan *et al.* 2001; Jezewski *et al.* 2007; Carlisle *et al.* 2009; MacCallum *et al.* 2009; Correia *et al.* 2010; Jiang *et al.* 2010; Mackie *et al.* 2016). It is possible that the combination of effects on these multiple pathways in the *grf10*Δ mutant led to strong attenuated virulence in mouse models of infection (Romanowski *et al.* 2012; Ghosh *et al.* 2015). The involvement of Grf10 in the copper response is novel, as this phenotype had not been reported in *S. cerevisiae*, providing new exploratory areas for orthologs of Grf10 in other fungal species. Since copper and iron availability lie at the center of the host–pathogen interface, Grf10 may play an essential role in sensing host nutrient levels and governing defenses against host nutrient immunity. Overall, our results add to the emerging concept that metabolism, morphogenesis, and nutritional immunity are intricately linked in *C. albicans* and that Grf10 participates in this transcriptional coordination.

## Supplementary Material

jkad070_Supplementary_Data

## Data Availability

Strains are listed in Table 1 and are available upon request. The RNA-seq data discussed in this publication have been deposited in NCBI's Gene Expression Omnibus (Edgar *et al.* 2002) and are accessible through GEO Series accession number GSE223218 (https://www.ncbi.nlm.nih.gov/geo/query/acc.cgi?acc=GSExxx). Supplementary materials are available at figshare (https://doi.org/10.25387/g3.14597676): Supplementary Tables 1 and 2 contain differential gene expression genome-wide at 1- and 4-h post-inoculation, respectively; Supplementary Tables 3 and 4 contain GO term analyses; and Supplementary Tables 5–7 contain differential gene expression in high copper conditions. Supplementary Figs. 1–5 contain growth data from cells challenged with FeCl_3_, CaCl_2_, MnCl_2_, ZnSO_4_, and CoCl_2_, respectively, over a range of concentrations; and Supplementary Fig. 6 shows papillae growth over time. The authors affirm that all data necessary for confirming the conclusions of the article are present within the article, figures, and tables.
