# Supplementary material for: Grf10 regulates the response to copper, iron, and phosphate in Candida albicans
Source: G3 (Bethesda). 2023 Mar 26;13(6):jkad070. doi: 10.1093/g3journal/jkad070 (PMC10234403; doi:10.1093/g3journal/jkad070)
Supplement: jkad070_Supplementary_Data [file jkad070_supplementary_data.zip › Supplemental Figure and Table Legends.docx]

**LEGENDS FOR SUPPLEMENTAL FIGURES S1-S6**

**Supplemental Figure S1.** Growth (OD_600_) of strains in a range of concentrations of CaCl_2_. Top row is the BWP17 background: the wild-type strain DAY286, *grf10*Δ mutant RAC117, and heterozygous restored strain RAC120. The second row is in the SN152 background: wild-type strains OHWT, *grf10*Δ mutant TF021, and heterozygous restored strain RAC256. Growth was compared in YPD medium or YPD medium supplemented with 200-1500 mM CaCl_2_, as indicated in the titles. YPD: wild-type in yellow; *grf10*Δ, light blue; restored, green; and YPD+ CaCl_2_: wild-type in dark blue; *grf10*Δ, orange; restored, gray. Graphs show the average OD_600_ from three biological replicates; the YPD controls are repeated in each graph for ease of comparison.

**Supplemental Figure S2.** Growth (OD_600_) of strains in a range of concentrations of CoCl_2_. Top row is the BWP17 background: the wild-type strain DAY286, *grf10*Δ mutant RAC117, and heterozygous restored strain RAC120. The second row is in the SN152 background: wild-type strains OHWT, *grf10*Δ mutant TF021, and heterozygous restored strain RAC256. Growth was compared in YPD medium or YPD medium supplemented with 1-5 mM CoCl_2_, as indicated in the titles. YPD: wild-type in yellow; *grf10*Δ, light blue; restored, green; and YPD+ CoCl_2_: wild-type in dark blue; *grf10*Δ, orange; restored, gray. Graphs show the average OD_600_ from three biological replicates; the YPD controls are repeated in each graph for ease of comparison.

**Supplemental Figure S3.** Growth (OD_600_) of strains in a range of concentrations of FeCl_3_, pH 4.0. Top row is the BWP17 background: the wild-type strain DAY286, *grf10*Δ mutant RAC117, and heterozygous restored strain RAC120. The second row is in the SN152 background: wild-type strains OHWT, *grf10*Δ mutant TF021, and heterozygous restored strain RAC256. Growth was compared in YPD medium or YPD medium supplemented with 0.3-1.5 mM FeCl_3_, as indicated in the titles. YPD: wild-type in yellow; *grf10*Δ, light blue; restored, green; and YPD+ FeCl_3_: wild-type in dark blue; *grf10*Δ, orange; restored, gray. Graphs show the average OD_600_ from three biological replicates; the YPD controls are repeated in each graph for ease of comparison.

**Supplemental Figure S4.** Growth (OD_600_) of strains in a range of concentrations of MnCl_2_. Top row is the BWP17 background: the wild-type strain DAY286, *grf10*Δ mutant RAC117, and heterozygous restored strain RAC120. The second row is in the SN152 background: wild-type strains OHWT, *grf10*Δ mutant TF021, and heterozygous restored strain RAC256. Growth was compared in YPD medium or YPD medium supplemented with 1-15 mM MnCl_2_, as indicated in the titles. YPD: wild-type in yellow; *grf10*Δ, light blue; restored, green; and YPD+ MnCl_2_: wild-type in dark blue; *grf10*Δ, orange; restored, gray. Graphs show the average OD_600_ from three biological replicates.

**Supplemental Figure S5.** Growth (OD_600_) of strains in a range of concentrations of ZnSO_4_. Top row is the BWP17 background: the wild-type strain DAY286, *grf10*Δ mutant RAC117, and heterozygous restored strain RAC120. The second row is in the SN152 background: wild-type strains OHWT, *grf10*Δ mutant TF021, and heterozygous restored strain RAC256. Growth was compared in YPD medium or YPD medium supplemented with 0.5-5 mM ZnSO_4_, as indicated in the titles. YPD: wild-type in yellow; *grf10*Δ, light blue; restored, green; and YPD+ ZnSO_4_: wild-type in dark blue; *grf10*Δ, orange; restored, gray. Graphs show the average OD_600_ from three biological replicates.

**Supplemental Figure S6.** Time course showing growth of wild-type strain DAY286 grown on YPD medium and YPD medium supplemented with 12 mM CuSO_4_. Pictures were taken each day for 8 days following spotting, as described in Materials and Methods. One of two biological replicates is shown; this experiment was done in parallel with the *grf10* Δ strain RAC117 and at 10 mM and 13 mM cuSO_4_ (not shown).

Supplemental Table S1. Differentially expressed genes at 1-hr post-inoculation, whole genome

Supplemental Table S2. Differentially expressed genes at 4-hr post-inoculation, whole genome

Supplemental Table S3. GO Term Analysis of the Differentially Expression Genes at 1-hr

Supplemental Table S4. GO Term Analysis of the Differentially Expression Genes at 4-hr

Supplemental Table S5. Differentially expressed genes from WT in YPD vs. 12 mM CuSO_4_

Supplemental Table S6. Differentially expressed genes from *grf10*Δ in YPD vs. 12 mM CuSO_4_

Supplemental Table S7. Differentially expressed *grf10*Δ vs. wild-type in high copper sulfate
