# Supplementary figures and images for: Grf10 regulates the response to copper, iron, and phosphate in Candida albicans
Source: G3 (Bethesda). 2023 Mar 26;13(6):jkad070. doi: 10.1093/g3journal/jkad070 (PMC10234403; doi:10.1093/g3journal/jkad070)

Figure S1

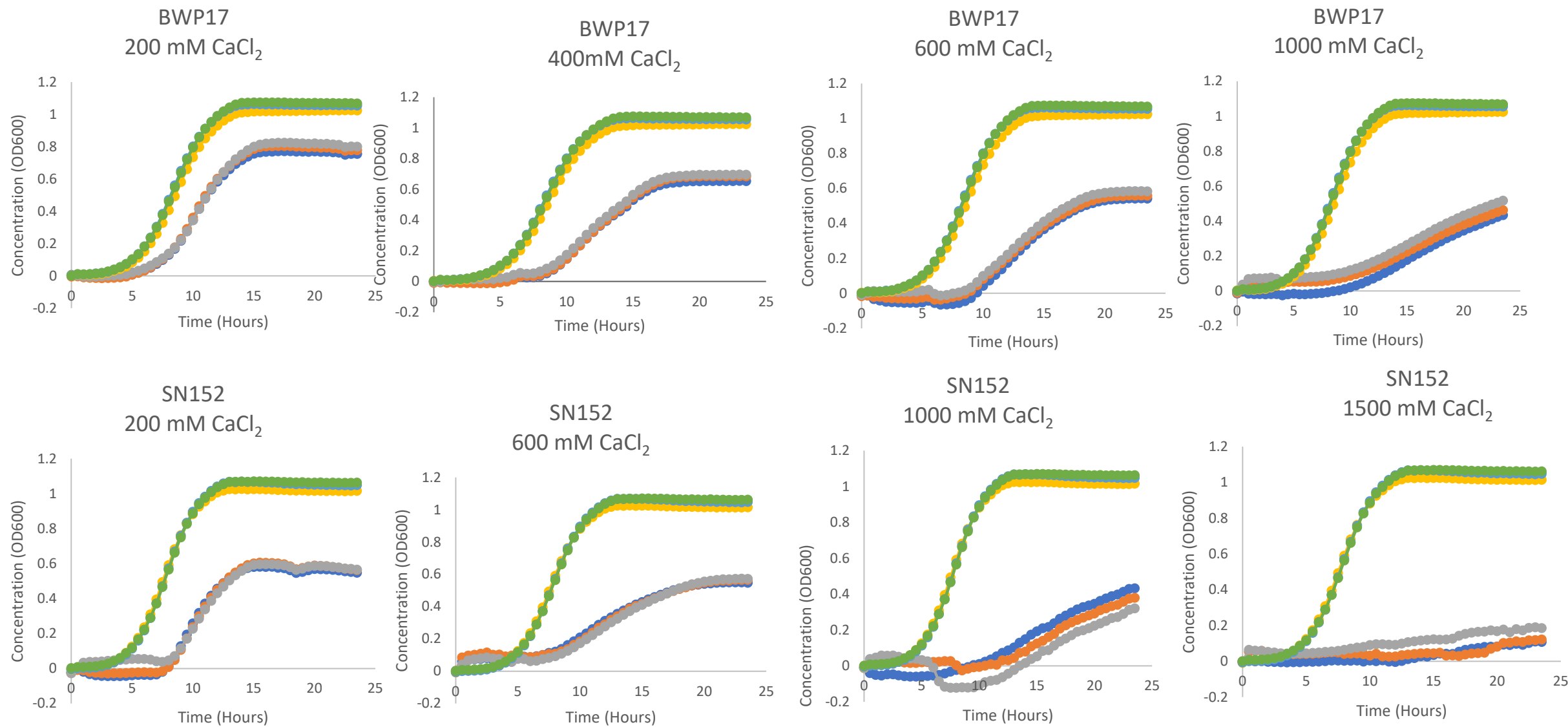

Figure S2

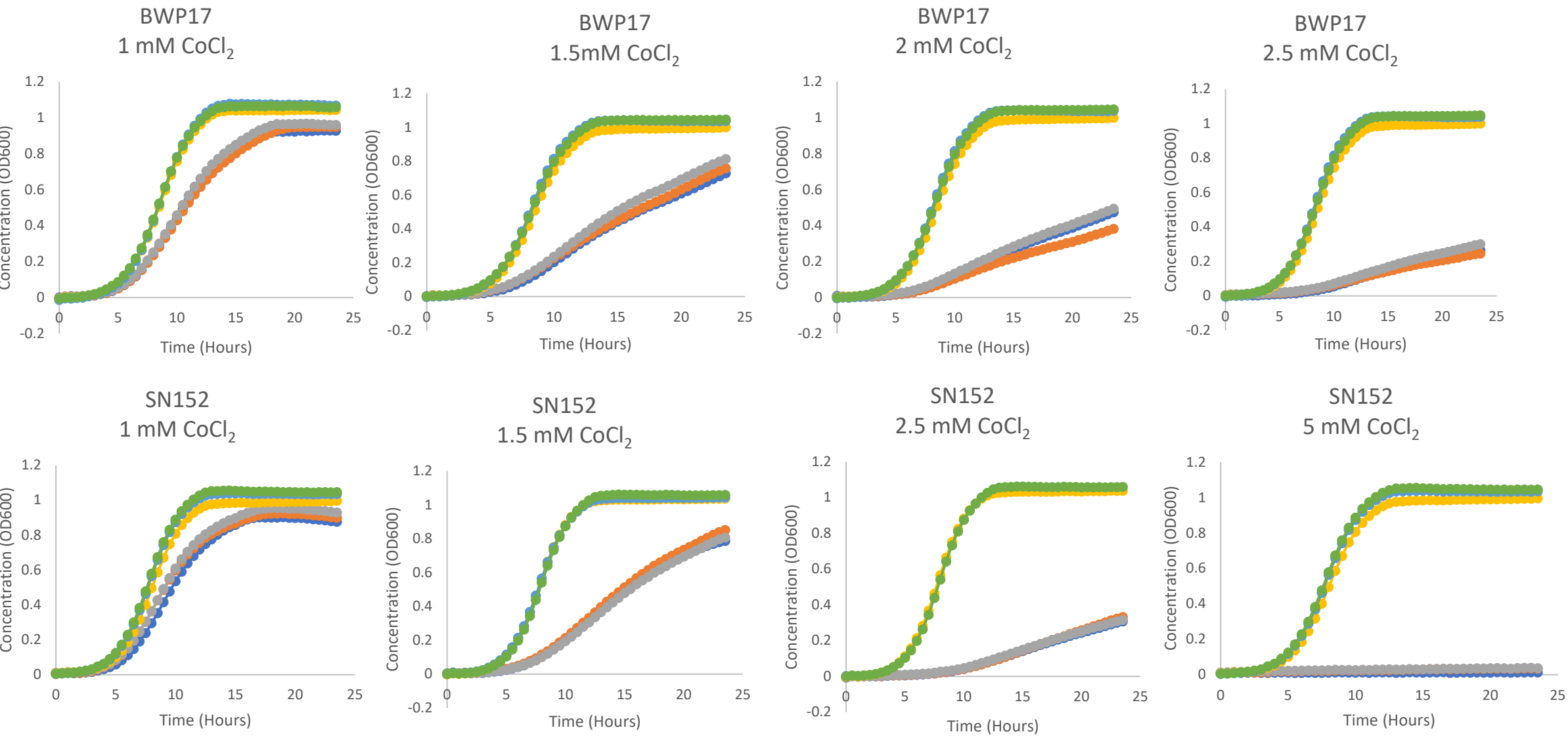

Figure S3

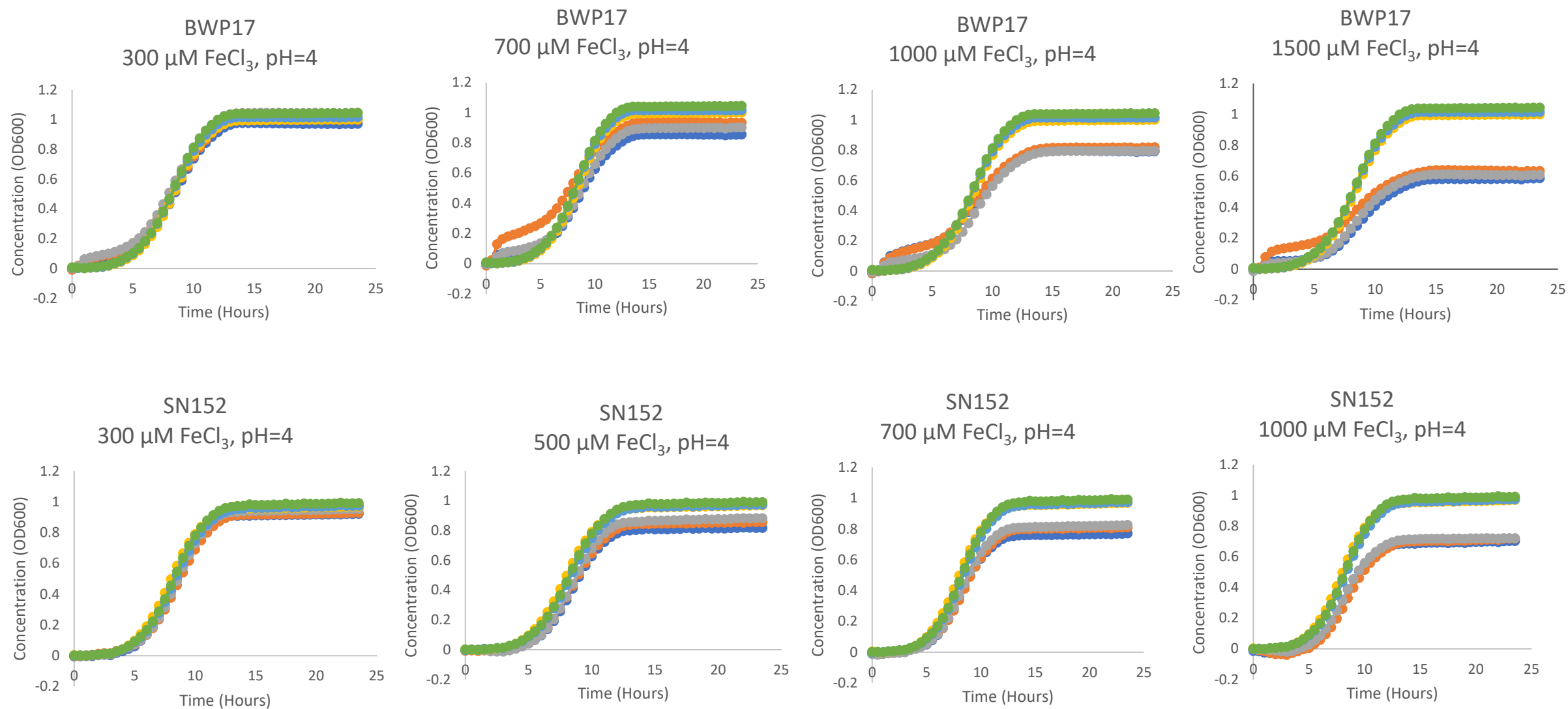

Figure S4

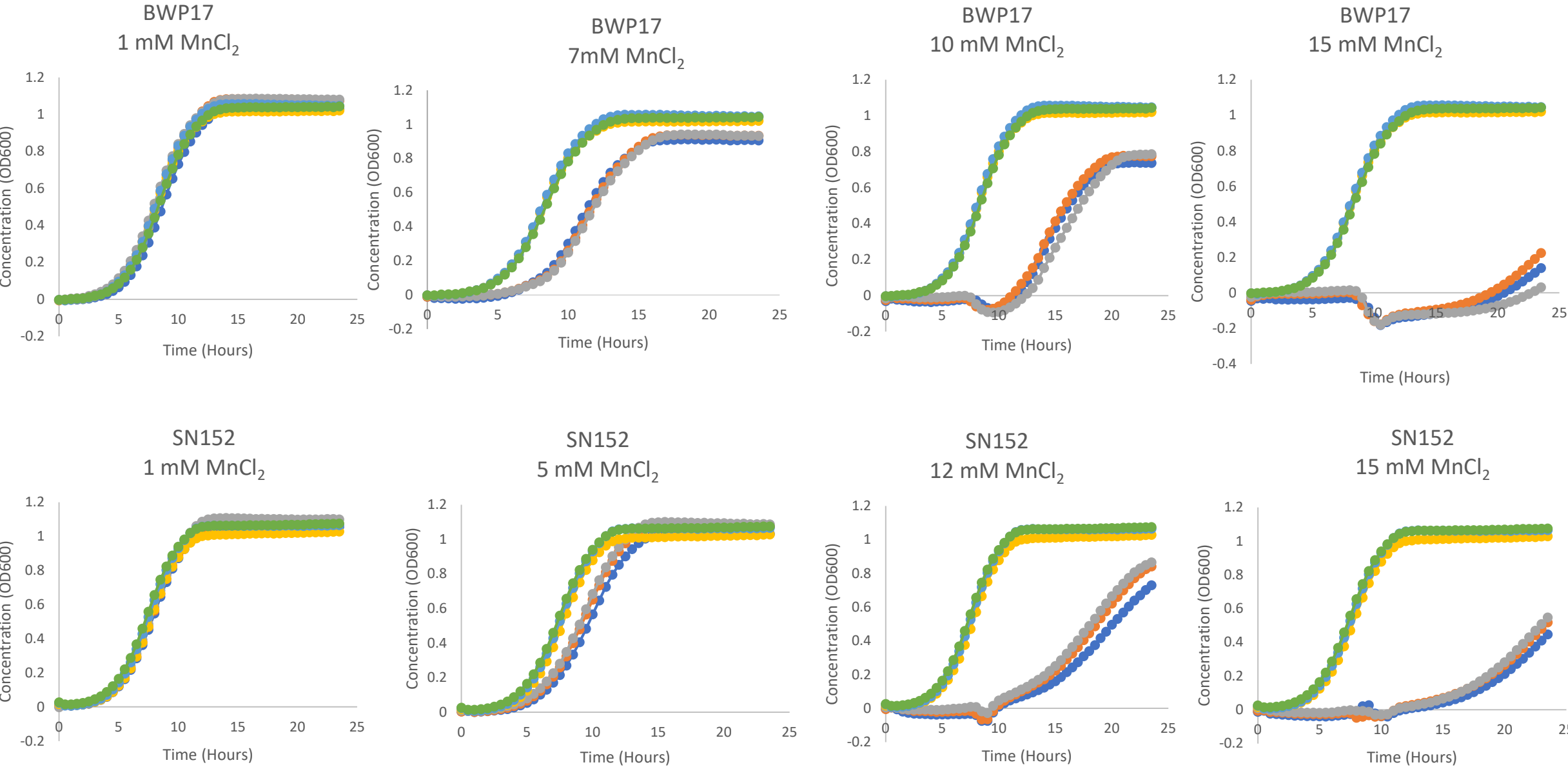

Figure S5

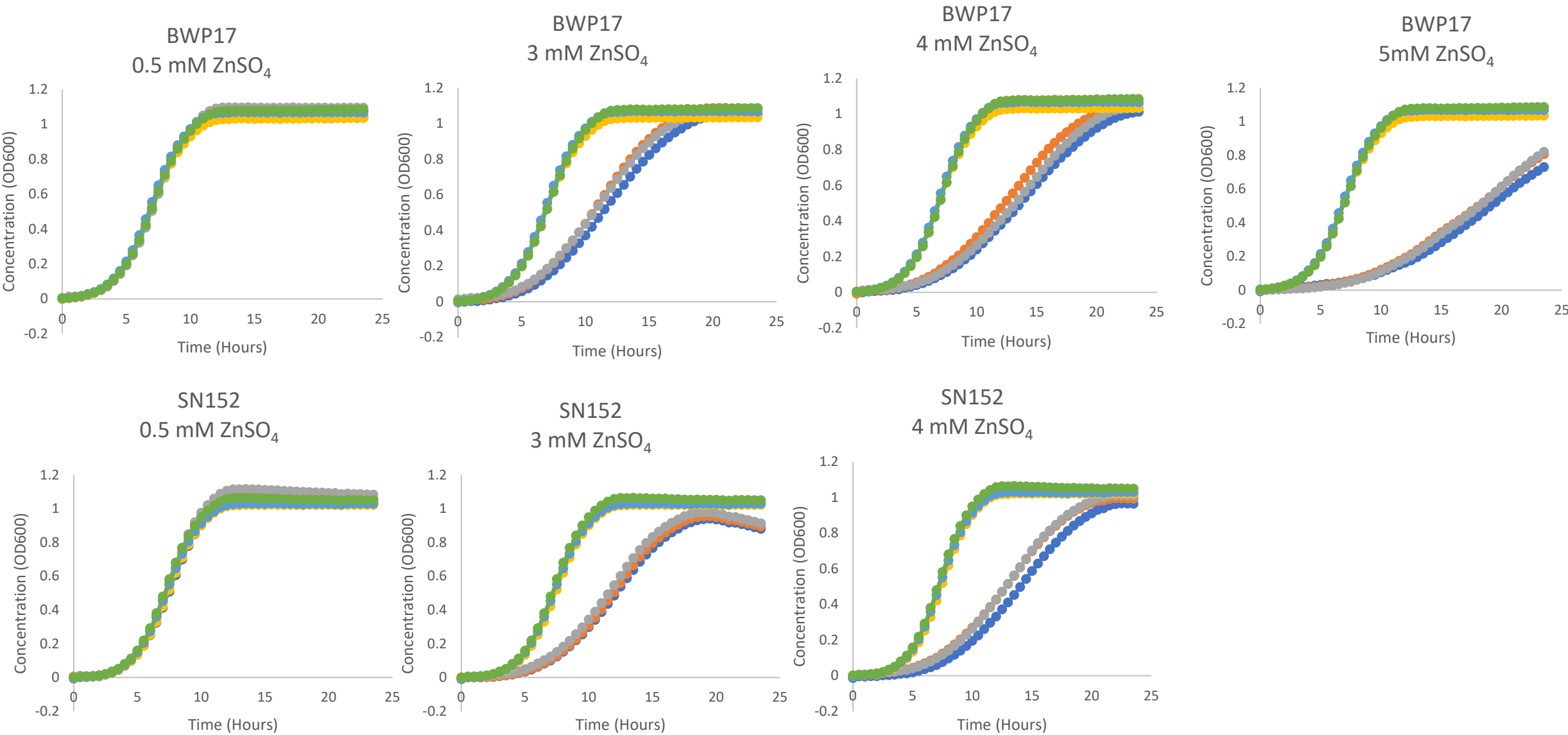

**Figure S6**

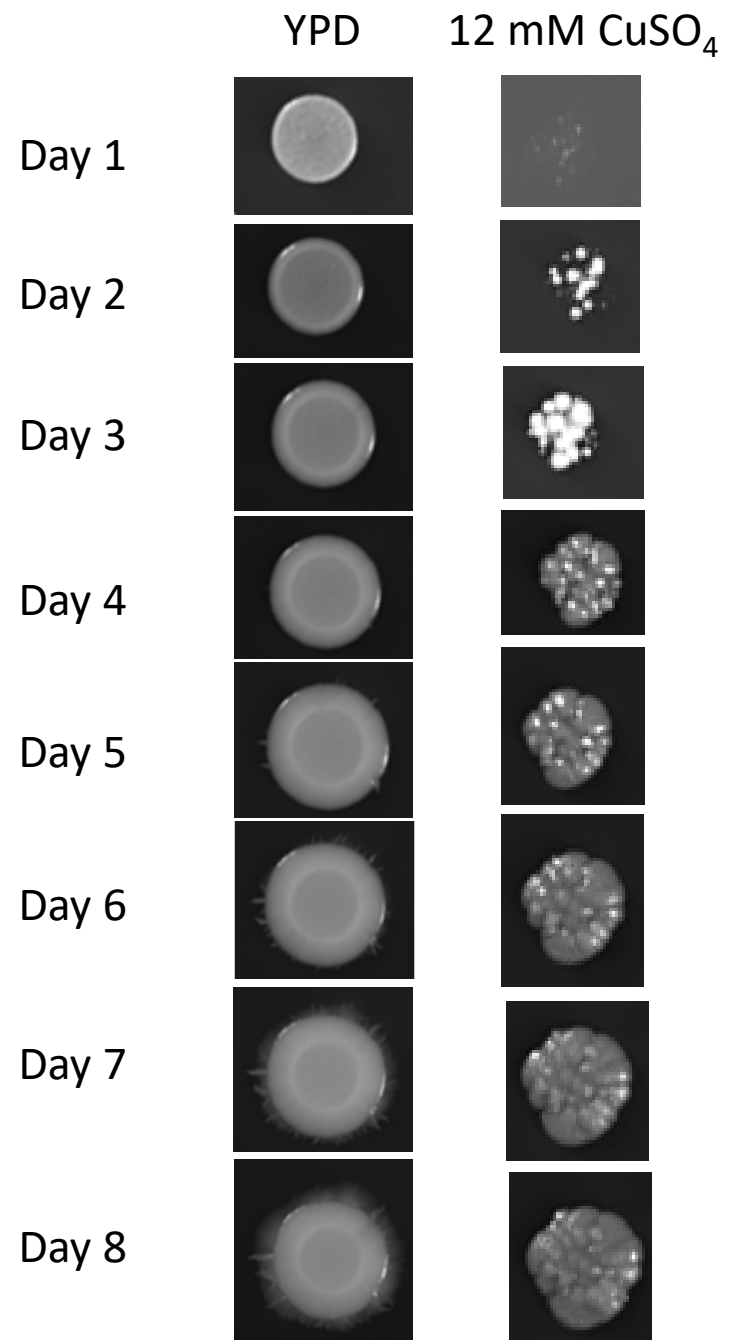

Supplement: jkad070_Supplementary_Data [file jkad070_supplementary_data.zip › Supplemental Figures.pdf]
